# Impact of dietary component clusters identified by K-means++ on renal function decline in a Taiwanese cohort

**DOI:** 10.1080/0886022X.2026.2667036

**Published:** 2026-06-01

**Authors:** Shang-Feng Tsai, Wei‑Ju Liu, Yu-Jung Lin, Chia-Lin Lee

**Affiliations:** a Department of Post-Baccalaureate Medicine, College of Medicine, National Chung Hsing University, Taichung, Taiwan; b Department of Internal Medicine, Division of Nephrology, Taichung Veterans General Hospital, Taichung, Taiwan; c Department of Digital Medicine, Clinical Informatics Division, Taichung Veterans General Hospital, Taichung, Taiwan; d Department of Medical Research, Intelligent Data Mining Laboratory, Taichung Veterans General Hospital, Taichung, Taiwan; e Department of Internal Medicine, Division of Endocrinology and Metabolism, Taichung Veterans General Hospital, Taichung, Taiwan; f Department of Digital Medicine, Division of Artificial Intelligence, Taichung Veterans General Hospital, Taichung, Taiwan; g Department of Medicine, School of Medicine, National Yang-Ming Chiao Tung University, Taipei, Taiwan

**Keywords:** Principal component analysis (PCA), K-means++ algorithm, diet pattern, renal function decline, estimated glomerular filtration rate (eGFR)

## Abstract

Dietary intake influences renal health, but the impact of overall dietary patterns on renal outcomes remains unclear. The K-means++ algorithm, which improves the stability of traditional K-means clustering, may provide a robust approach for identifying real-world dietary behaviors. We analyzed 24,820 adults from the MJ Health Research Database in Taiwan. Dietary intake was assessed using a validated food frequency questionnaire, and patterns were derived with the K-means++ algorithm. Three clusters – non-healthy, normal, and healthy – were identified at baseline and follow-up. Renal outcomes were evaluated by estimated glomerular filtration rate (eGFR), with worsening defined as an annual decline >1 mL/min/1.73 m^2^. At baseline, three distinct dietary clusters were identified. The non-healthy cluster was characterized by high intake of sugar-sweetened beverages, processed foods, and condiments, whereas the healthy cluster was characterized by greater consumption of nutrient-rich foods such as dairy, eggs, beans, and vegetables. Over follow-up, participants transitioning from a non-healthy to a healthy cluster exhibited a modestly lower risk of renal function worsening (adjusted odds ratio = 0.81, 95% CI: 0.67–0.99, *p* = 0.042). The K-means++ algorithm effectively identified meaningful dietary patterns and revealed clinically relevant associations with renal outcomes. These findings suggest that dietary modification may contribute to renal health preservation and demonstrate that the principal component analysis (PCA)-based clustering approach with K-means++ initialization provides a stable and appropriate framework for identifying dietary patterns in nutritional epidemiology.

## Introduction

Dietary intake plays a pivotal role in maintaining renal health and modulating the progression of chronic kidney disease (CKD). Accumulating evidence indicates that dietary patterns rich in fruits, vegetables, whole grains, and low-fat dairy products are associated with slower renal function decline, whereas high consumption of processed foods, red meat, and sugar-sweetened beverages is linked to an increased risk of CKD onset and progression [1–3]. Several large cohort studies have demonstrated that adherence to healthy dietary patterns, such as the Dietary Approaches to Stop Hypertension (DASH) or Mediterranean diet, is associated with lower incidence of CKD and reduced all-cause mortality among patients with impaired renal function [4,5]. Conversely, unhealthy dietary habits that promote metabolic disturbances, including high sodium and phosphorus intake, have been shown to exacerbate albuminuria, hypertension, and accelerated estimated glomerular filtration rate (eGFR) decline [6,7]. These findings underscore the importance of understanding dietary intake patterns when investigating risk factors and preventive strategies for deterioration of renal function.

In the field of nutrition, it is challenging to isolate the effects of individual foods or nutrients on kidney outcomes, as patients consume diverse combinations of foods that may interact in complex ways. Single-nutrient approaches often fail to capture the synergistic or antagonistic relationships among dietary components, leading to inconsistent or limited finding [8–10]. Consequently, recent research has increasingly shifted toward examining overall dietary patterns using statistical methods such as principal component analysis (PCA) [11,12], reduced rank regression (RRR) [13], or clustering algorithms [14], which better reflect real-world eating behaviors and their associations with renal outcomes [15]. Nevertheless, research on the association between dietary patterns and renal function is still limited.

K-means clustering is an unsupervised machine learning algorithm used to group data into a pre-specified number of clusters (denoted by *k*). The goal is to partition observations into clusters such that data points within the same cluster are as similar as possible to each other, while being as different as possible from points in other clusters. K-means clustering was employed to characterize transient dietary patterns [16–19]. Although widely used, the K-means algorithm has notable limitations. It requires predefining the number of clusters, is sensitive to initial centroid placement, and assumes clusters are spherical and of similar size. Moreover, it is affected by outliers and relies on hard assignments, which may oversimplify complex dietary or clinical patterns [20,21]. K-means++ is an enhanced initialization method for K-means clustering that selects initial centroids in a probabilistic manner, favoring points farther apart, thereby improving cluster stability, reducing sensitivity to random starting values, and accelerating convergence while maintaining computational efficiency [22]. To date, only one study has applied the K-means++ algorithm to cluster dietary patterns [23]; however, no validation of these clusters against clinical outcomes, such as renal function, has been reported in the current literature.

Therefore, in this study, we applied the K-means++ algorithm as a robust clustering approach combining PCA and K-means++ initialization to identify dietary intake patterns and further investigated the association of these K-means++-derived dietary patterns with renal outcomes.

## Materials and methods

### Study population and data collection

Data were obtained from the MAJOR (MJ) Health Research Database, a nationwide prospective cohort established by the MJ Health Management Institution in Taiwan (www.mjclinic.com.tw) [24]. Since 1994, the MJ cohort has enrolled approximately 600,000 adults who voluntarily participated in standardized health screening programs. Detailed descriptions of the study design and data collection procedures have been reported previously. At enrollment, participants completed a standardized health assessment, including a structured questionnaire, physical examination, and fasting laboratory tests, all performed under International Organization for Standardization (ISO) 9001-certified protocols. Annual follow-up evaluations were conducted, and data were systematically updated by the MJ Health Research Foundation to ensure accuracy and longitudinal completeness.

For the present analysis, inclusion criteria were as follows: (1) completion of at least five follow-up visits within an eight-year observation period (January 1998–December 2013); (2) availability of at least five recorded eGFR values during this period; (3) baseline eGFR ≥60 mL/min/1.73 m^2^; and (4) completion of the comprehensive nutrition questionnaire at both baseline and follow-up. Renal function decline was defined as mean annual reduction in eGFR. After applying these criteria, 24,820 participants were included in the final analysis (Figure S1). Baseline demographic and clinical characteristics are summarized in Table S1.

This study was approved by the Institutional Review Board of Taichung Veterans General Hospital, Taiwan (IRB No. CE18312A). All procedures complied with relevant ethical standards and regulations, and written informed consent was obtained from all participants or their legal guardians, as appropriate. This study was conducted in accordance with the Declaration of Helsinki.

### Dietary assessment by semi-quantitative food frequency questionnaire

Dietary patterns were evaluated using a semi-quantitative food frequency questionnaire (FFQ) that had been standardized and validated in prior studies conducted by the MJ Health Research Foundation [25,26]. The FFQ was specifically developed to capture habitual dietary intake among the Taiwanese population and initially comprised of 22 food groups, each with predefined portion sizes and graded frequency categories across five levels, ranging from the lowest to the highest intake, expressed as servings per day or per week. For instance, the consumption of beans and bean products was categorized as none or <1 serving per week, 1–3 servings per week, 4–6 servings per week, 1 serving per day, or ≥2 servings per day, with one serving defined as one standard tofu block, one cup of soymilk, or two pieces of dried tofu.

For longitudinal comparability, only dietary items consistently assessed at both baseline and follow-up were retained. Thus, the original 22 FFQ food groups were reduced to 17 for clustering analyses. Reported intake frequencies were converted into average weekly servings and standardized to generate comparable feature variables. Participants with missing data for any of the 17 items were excluded.

### Dietary pattern assessment by principal component analysis and K-means++

PCA is a powerful non-parametric linear dimensionality reduction technique. Its main goal is to simplify a complex, high-dimensional dataset by transforming it into a smaller set of new, uncorrelated variables called principal components (PCs). PCA achieves this by finding a new axis system that retains the maximum possible variance (information) of the original data, thereby helping with visualization, memory efficiency, and speeding up machine learning algorithms [27,28]. The number of PCs retained was determined based on eigenvalues greater than 1.0, inspection of the scree plot, and the cumulative proportion of explained variance to ensure that the selected components captured the majority of the variability in the dietary variables. Unsupervised clustering was performed using the K-means algorithm, with cluster initialization based on the K-means++ method to improve stability across multiple random starts [22,29]. Euclidean distance was applied as a similar metric. To identify the optimal number of clusters, we conducted a grid search for *k* values ranging from 2 to 8 and assessed internal validity using the silhouette coefficient, defined as:

$$s\left(i\right)=\frac{b\left(i\right)-a\left(i\right)}{\text{max}\left\{a\left(i\right),b\left(i\right)\right\}}$$

Here, *a*(*i*) represents the average distance between sample *i* and all other samples within the same cluster, while *b*(*i*) denotes the average distance between sample *i* and samples in the nearest neighboring cluster. The overall mean silhouette value *s*(*i*) was used to determine the optimal number of clusters (*k*), with higher values indicating better separation. The final number of clusters was determined by considering both the maximum average silhouette coefficient and the elbow point identified from the within-cluster sum of squares (WCSS) curve, ensuring that the selected clustering solution achieved both statistical validity and interpretability of dietary patterns. Additionally, to ensure that the selected number of clusters was analytically appropriate, we evaluated the WCSS and applied the elbow method. These approaches were used to determine the optimal number of distinct dietary pattern groups.

To visualize cluster separation, PCA was applied to project the clustering features onto a two-dimensional space, in which the centroids derived from the K-means++ algorithm were also plotted. After clustering, each cluster was further labeled according to the characteristic dietary patterns observed within that group (such as non-health, normal, and health). The non-healthy diet pattern was characterized by a high intake the following food, such as sugar-sweetened beverages and a greater consumption of processed foods, instant noodles, and condiments, along with relatively lower intake of nutrient-rich foods such as milk, dairy products, eggs, beans and bean products, and fish/seafood. In contrast, the healthy diet pattern was marked by higher consumption of nutrient-dense foods and a lower intake of sugary drinks and processed items. The normal diet pattern represented an intermediate dietary behavior, falling between the non-healthy and healthy diet patterns.

### Kidney function assessment

Kidney function was evaluated using the Chronic Kidney Disease Epidemiology Collaboration (CKD-EPI) equation [30]: 141 × min (Scr/*κ*, 1)*^α^* × max (Scr/*κ*, 1)^−1.209^ × 0.993^Age^ × 1.018 (if female) × 1.159 (if black) where Scr is serum creatinine (mg/dL), *κ* (kappa) is 0.7 for females and 0.9 for males, and *α* (alpha) is −0.329 for females and −0.411 for males. The functions min and max denote the minimum and maximum of Scr/*κ* or 1, respectively.

To estimate longitudinal changes in renal function, we applied a generalized estimating equation (GEE) model with an exchangeable working correlation structure to account for within-subject correlations of repeated measures. The dependent variable was eGFR (mL/min/1.73 m^2^), repeatedly measured for each participant, with elapsed time (years) since baseline as the primary predictor. The model was specified as:

$${\text{eGFR}}_{\text{ij}}=\beta 0+\beta 1\times \left(\Delta t_{\text{ij}}\right)+\varepsilon_{\text{ij}}$$

where CKD-EPI ${\text{eGFR}}_{\text{ij}}$ is the eGFR value for subject *i* at the *j*th visit, $\Delta t_{\text{ij}}$ is the time interval (years) since baseline, *β*0 is the intercept representing baseline eGFR, *β*1 is the slope reflecting the mean annual change in eGFR, and $\varepsilon_{\text{ij}}$ is the error term, assumed to follow a normal distribution with exchangeable correlation within subjects.

Because eGFR physiologically declines with aging – at an average rate of approximately 1 mL/min/1.73 m^2^ per year due to gradual nephron loss, this value is widely considered the upper limit of normal age-related decline [31,32] – this rate was adopted as the reference. For the purpose of this study, we defined abnormal renal function decline operationally as an annual eGFR decrease greater than −1 mL/min/1.73 m^2^. In addition, we conducted sensitivity analyses using clinically relevant eGFR endpoints, including an annual decline of >5 mL/min/1.73 m^2^/year or a greater than 30% decline from baseline.

### Statistical analyses

All statistical analyses were performed using SAS version 9.4 (SAS Institute, Cary, NC), R version 4.2.1 (R Foundation for Statistical Computing, Vienna, Austria), and Python version 3.9.7 (Python Software Foundation, Wilmington, DE). Continuous variables are presented as mean ± standard deviation (SD), and categorical variables as counts with percentages.

Differences in mean eGFR levels and annual rates of eGFR change across dietary clusters were assessed using analysis of variance (ANOVA) and generalized linear models (GLMs). To further evaluate the association between dietary clusters and renal function decline, Cox proportional hazards regression analyses were conducted. Odds ratio (OR) with 95% confidence intervals (CIs) were estimated to compare the risk of renal function progression across dietary clusters, using the Health to Health as the reference group. Multivariable models were adjusted for blood pressure, body mass index (BMI), blood glucose, and baseline eGFR. We also performed sensitivity analyses using models further adjusted for age, sex, or both. Statistical significance was defined as a two-sided *p* value <0.05.

## Results

### Baseline characteristics of the cohort

In the final cohort, a total of 24,820 participants were included (Supplementary Figure 1). The cohort was relatively young, with a mean age of 37.8 ± 11.5 years, and exhibited a normal BMI (22.9 ± 3.5 kg/m^2^) (Supplementary Table 1). The prevalence of metabolic risk factors was low, with 3.1% having diabetes mellitus and 9.2% hypertension. Mean SBP and LDL-C levels were 118 ± 16 mmHg and 116 ± 32 mg/dL, respectively. Renal function was well preserved, with an eGFR of 92.7 ± 16.0 mL/min/1.73 m^2^. In Supplementary Figures 2 and 3, we observed that cluster compactness was most analytically meaningful when *k* = 3 at both baseline and follow-up. Moreover, the three-cluster solution demonstrated strong theoretical interpretability and practical relevance within the context of this study. Therefore, we selected *k* = 3 as the optimal number of clusters.

### Results of dietary pattern clusters by K-means++ at baseline

**Figure 1.**
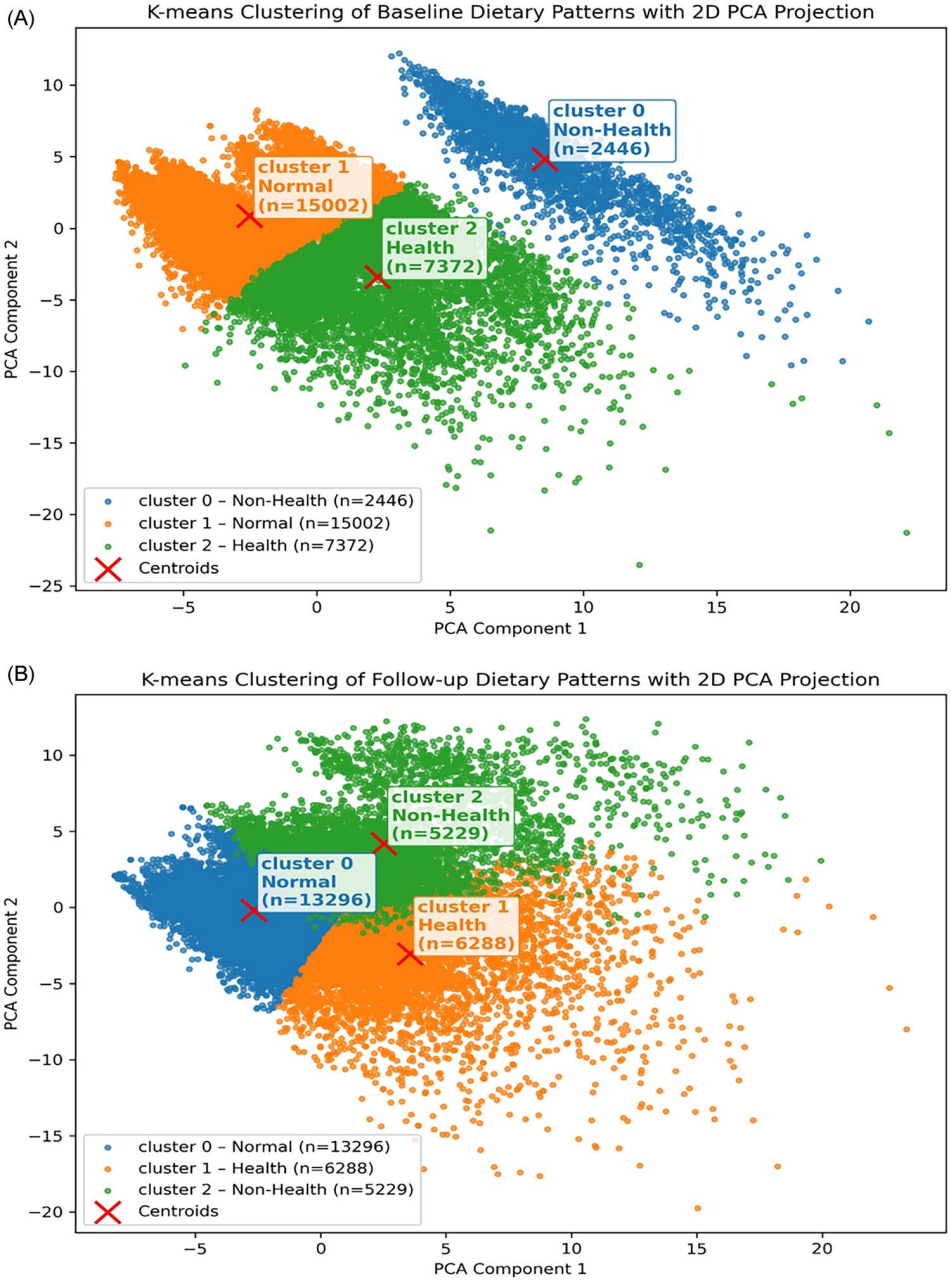
K-means++ clustering of dietary patterns at (A) baseline and (B) follow-up, visualized using 2D principal component analysis (PCA) projection. Participants were classified into three clusters: Non-Health, Health, and Normal. (A) K means++ clustering of baseline dietary patterns with 2D PCA projection. (B) K-means++ clustering of follow-up dietary patterns with 2D PCA projection.

At baseline, three distinct dietary clusters were identified among the 24,820 participants: cluster 0 (Non-Health, *n* = 2,446), cluster 1 (Normal, *n* = 15,002), and cluster 2 (Health, *n* = 7,372) (Figure 1(A); Supplementary Table 2). Participants in the Health cluster (cluster 2) demonstrated the highest consumption of nutrient-rich foods, including milk (2.75 ± 2.93 servings/week), dairy products (1.65 ± 1.77), eggs (4.70 ± 2.49), beans and bean products (3.97 ± 2.67), fish/seafood (4.33 ± 2.70), whole grains (2.83 ± 3.47), fruits (2.30 ± 2.03), and both light- and dark-colored vegetables (3.60 ± 2.70 and 3.35 ± 2.54, respectively) compared with the other clusters (all *p* < 0.001).

By contrast, the Non-Health cluster (cluster 0) was characterized by extremely high intake of sugary drinks (14.00 ± 0.00 servings/week), as well as greater consumption of processed foods, instant noodles, condiments, and organ meats relative to the Normal cluster (*p* < 0.001 for all). This group also showed high intake of staple foods, meat, and condiments, but relatively lower consumption of fruits and vegetables. The Normal cluster (cluster 1) generally represented moderate intake patterns across most food categories, with lower frequencies of both unhealthy and highly nutritious foods.

Overall, dietary clusters were significantly differentiated across all food categories (*p* < 0.001), with a clear trend toward healthier food choices in the Health cluster and higher intake of processed and sugary items in the Non-Health cluster

### Results of dietary pattern clusters by K-means++ at follow-up

At the end of follow-up, three dietary clusters remained evident among the 24,820 participants (cluster 2: Non-Health, *n* = 5,229; cluster 0: Normal, *n* = 13,296; cluster 1: Health, *n* = 6,288) (Figure 1(B); Supplementary Table 3). The Normal cluster (cluster 1) exhibited the most favorable dietary composition, with the highest intake of dairy products (1.87 ± 1.83 servings/week), beans and bean products (4.61 ± 2.85), fish/seafood (4.18 ± 2.51), whole grains (3.62 ± 3.57), fruits (2.82 ± 2.12), and both light- and dark-colored vegetables (4.91 ± 2.86 and 4.71 ± 2.81, respectively).

The Non-Health cluster (cluster 2) was characterized by persistently high consumption of sugary drinks (7.92 ± 3.27 servings/week), meat (5.55 ± 3.10), condiments (1.99 ± 2.00), and organ meats (1.05 ± 1.03), but comparatively lower intake of protective foods such as vegetables, fruits, and whole grains. In contrast, the normal cluster (cluster 0) demonstrated relatively higher consumption of staple foods, but had the lowest intake of dairy products, eggs, beans, fish, and vegetables. Notably, this group also reported the lowest intake of sugary drinks (1.40 ± 1.24 servings/week) and processed foods (0.90 ± 0.79). Overall, the dietary clusters at follow-up showed significant differences across all food categories (*p* < 0.001), highlighting a shift toward greater heterogeneity in dietary behaviors over time.

### Results of annual eGFR decline by dietary clusters

**Figure 2.**
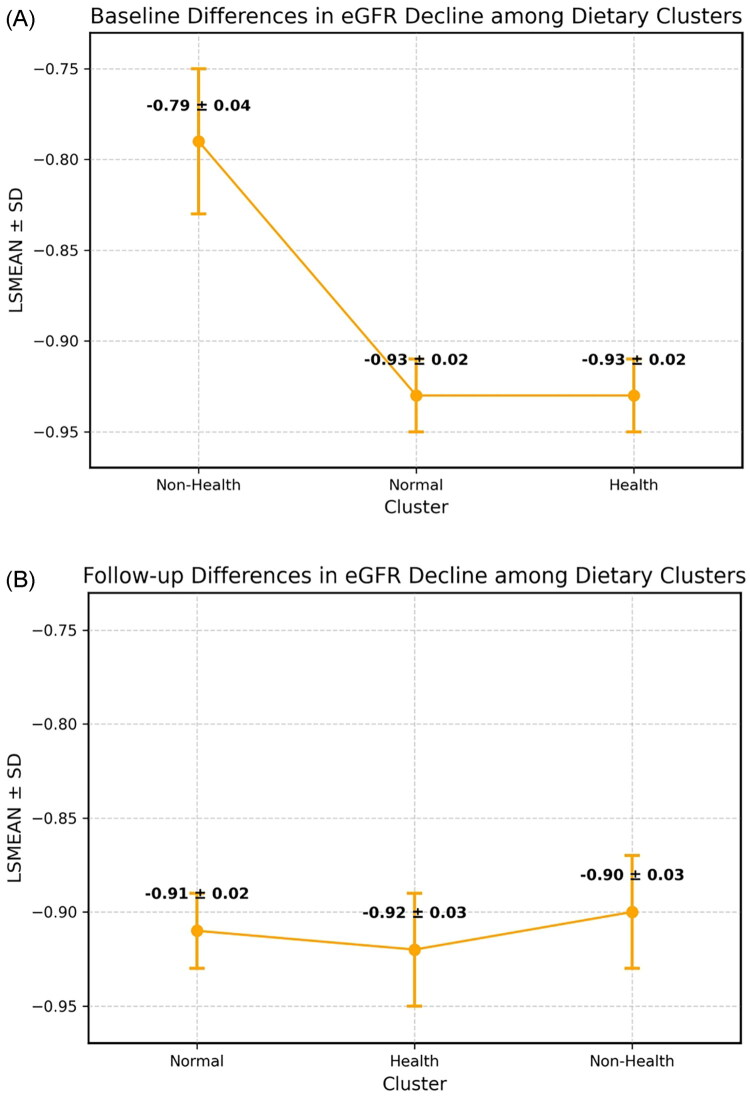
Annual eGFR decline according to dietary clusters at (A) baseline and (B) follow-up. Error bars indicate standard deviation. Abbreviations: LSMEAN, least squares mean; SD, standard deviation; eGFR, estimated glomerular filtration rate.

Figure 2 illustrates the annual decline in eGFR across dietary clusters at both baseline (panel A) and follow-up (panel B). At baseline, participants in the Normal and Health clusters showed slightly greater mean eGFR declines (0.93 ± 0.02 and 0.93 ± 0.02 mL/min/1.73 m^2^ per year, respectively) compared with the Non-Health cluster (0.79 ± 0.04 mL/min/1.73 m^2^ per year). At follow-up, however, the magnitude of decline was more uniform across clusters, with least squares mean (LSmean) annual declines of 0.90 ± 0.03 in the Non-Health cluster, 0.90 ± 0.03 in the Normal cluster, and 0.91 ± 0.02 in the Health cluster. These findings indicate that while dietary clusters were associated with different baseline trajectories, longitudinal differences in eGFR decline converged during follow-up.

### Results of annual renal function decline across dietary clusters stratified by baseline eGFR

At baseline, 24,820 participants were classified into three dietary clusters: cluster 0 (Non-Health, *n* = 2,446, 9.9%), cluster 1 (Normal, *n* = 15,002, 60.4%), and cluster 2 (Health, *n* = 7,372, 29.7%) (Table 1). Across all clusters, the mean annual eGFR decline was less than 1 mL/min/1.73 m^2^. Specifically, the LSmean decline was 0.79 ± 0.04 for the Non-Health cluster, 0.93 ± 0.02 for the Normal cluster, and 0.93 ± 0.02 for the Health cluster. Stratification by baseline eGFR demonstrated consistent trends. Among participants with eGFR >90 mL/min/1.73 m^2^, annual decline was most pronounced: 1.47 ± 0.06 in the Non-Health cluster, 1.65 ± 0.02 in the Normal cluster, and 1.59 ± 0.03 in the Health cluster. In contrast, those with baseline eGFR 60–90 had markedly smaller declines, ranging from 0.06 ± 0.05 in the Non-Health cluster to 0.14 ± 0.02 in the Normal cluster.

**Table 1.** Distribution of participants and annual eGFR decline across dietary clusters at baseline and follow-up.

Time	Baseline						Follow-up					
eGFR	60+		60–90		>90		60+		60–90		>90	
Cluster	*n* (%)	LSmean ± SD	*n* (%)	LSmean ± SD	*n* (%)	LSmean ± SD	*n* (%)	LSmean ± SD	*n* (%)	LSmean ± SD	*n* (%)	LSmean ± SD
Cluster 0 (Non-Health)	2,446 (9.9)	0.79 ± 0.04	1,181 (10.1)	0.06 ± 0.05	1,265 (9.6)	1.47 ± 0.06	5,229 (21.1)	0.92 ± 0.03	2,284 (19.6)	0.01 ± 0.04	2,945 (22.4)	1.59 ± 0.04
Cluster 1 (Normal)	15,002 (60.4)	0.93 ± 0.02	7,226 (62.0)	0.14 ± 0.02	7,776 (59.1)	1.65 ± 0.02	13,296 (53.6)	0.90 ± 0.03	6,489 (55.7)	0.16 ± 0.02	6,807 (51.7)	1.64 ± 0.03
Cluster 2 (Health)	7,372 (29.7)	0.93 ± 0.02	3,251 (27.9)	0.10 ± 0.03	4,121 (31.3)	1.59 ± 0.03	6,288 (25.3)	0.91 ± 0.02	2,879 (24.7)	0.13 ± 0.03	3,409 (25.9)	1.60 ± 0.04

LSmean: least squares mean; SD: standard deviation; eGFR: estimated glomerular filtration rate.

At follow-up, the distribution of participants shifted substantially. The proportion classified as Non-Health increased to 21.1% (*n* = 5,229), while Normal and Health clusters accounted for 53.6% (*n* = 13,296) and 25.3% (*n* = 6,288), respectively. Annual eGFR declines were similar across clusters, with LSmeans of 0.90 ± 0.03 (Non-Health), 0.90 ± 0.03 (Normal), and 0.91 ± 0.02 (Health). Participants with preserved baseline renal function (eGFR >90) again showed the steepest decline: 1.59 ± 0.04 in Non-Health, 1.64 ± 0.03 in Normal, and 1.60 ± 0.04 in Health clusters. Overall, dietary clusters were associated with different baseline and follow-up distributions, but the patterns of eGFR decline were broadly comparable across groups, with greater declines observed in participants starting with higher baseline eGFR.

In Supplementary Figure 4, to further ensure clarity and consistency, we added visual summaries of food group intake profiles for all three clusters at baseline and follow-up (Figures S4A and S4B). As illustrated in these figures, the Non-Health cluster consistently demonstrated the highest intake of sugary beverages, sweets, and processed foods at both time points, whereas the Health cluster exhibited greater consumption of nutrient-dense food groups.

### Risk of renal function worsening (annual eGFR decline >1 mL/min/1.73 m^2^) across dietary cluster transitions

**Figure 3.**
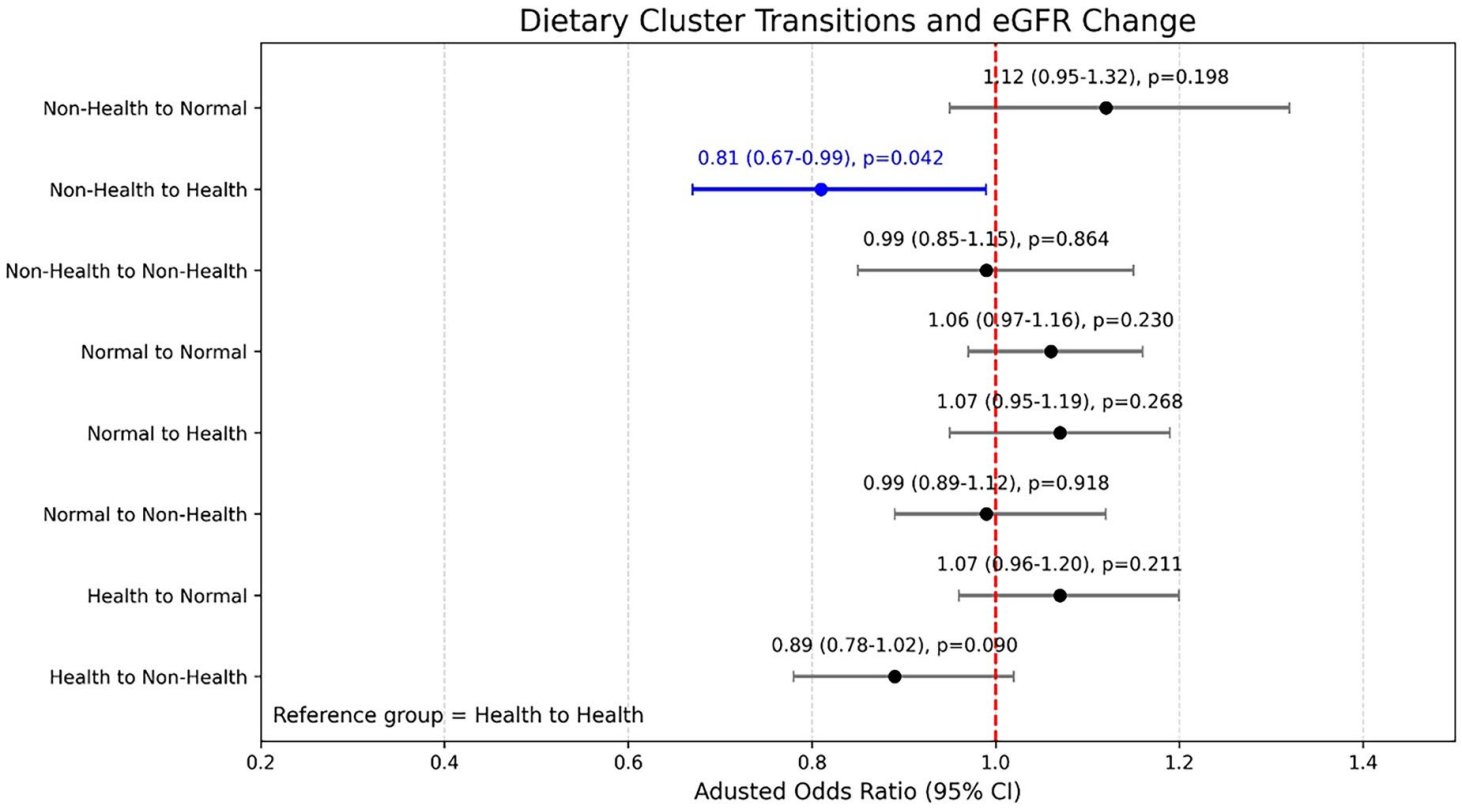
Adjusted odds ratios (AORs) with 95% CIs for renal function worsening (annual eGFR decline >1 mL/min/1.73 m^2^) by dietary cluster transitions. The reference group was participants who remained in the health cluster. Adjustment for body mass index (BMI), blood pressure, blood glucose, and baseline eGFR at both baseline and follow-up, as well as baseline eGFR. Dietary pattern transitions were defined based on changes in cluster membership between baseline and follow-up assessments. Participants were categorized into nine groups reflecting all possible transitions among Non-Health, Normal, and Health clusters (e.g., Non-Health to Health, Health to Non-Health, etc.). The Health to Health group, representing individuals who consistently maintained a healthy dietary pattern, was used as the reference category.

Figure 3 presents the AOR (adjusted OR) and 95% CIs for renal function decline according to transitions in dietary patterns. Using participants who consistently remained in the Health cluster as the reference group, participants who transitioned from a non-healthy to a healthy diet pattern had a significantly lower risk of renal function worsening, defined as an annual eGFR decline >1 mL/min/1.73 m^2^ (AOR = 0.81, 95% CI: 0.67–0.99, *p* = 0.042). Otherwise, no other food cluster transition shows any significant difference in the risk of worsening renal function.

Sensitivity analyses with additional adjustments for sex, age, or both are presented in Supplementary Figure 5 (A: sex, B: age, C: both age and sex). The results of these supplementary models were directionally consistent with the main analysis, although the effect estimates were attenuated, suggesting that part of the observed association may be attributable to age-related physiological decline in kidney function.

Another sensitivity analysis using alternative endpoints of renal function deterioration is presented in Supplementary Figure 6 (A: annual eGFR decline >5 mL/min/1.73 m^2^; B: >30% decline in eGFR from baseline). When applying these stricter definitions of renal decline, the ‘Non-Health to Health’ dietary transition continued to demonstrate a lower risk, although the associations did not reach statistical significance (AOR = 0.71 and AOR = 0.85, respectively). Across all models, the direction of the effect remained consistent, supporting the robustness of the primary findings.

## Discussion

In this study, we applied the K-means++ algorithm to cluster dietary intake patterns and examined their association with renal function outcomes. Using K-means++, three distinct dietary clusters were identified: a healthy pattern, characterized by the highest consumption of nutrient-rich foods such as milk, dairy products, and eggs; a non-healthy pattern, characterized by greater intake of sugary drinks, processed foods, instant noodles, condiments, and organ meats; and a normal pattern, representing moderate or balanced intake between these two extremes. Furthermore, transitions from non-healthy to healthy dietary patterns, as defined by K-means++ clustering, were associated with a reduced risk of renal function worsening, defined as an annual eGFR decline >1 mL/min/1.73 m^2^. Although the magnitude of annual eGFR differences observed between dietary transition groups was modest at the individual level, even small differences in the rate of renal decline may translate into substantial reductions in CKD burden at the population level, particularly in relatively young cohorts with long-term follow-up potential.

Clustering approaches are widely used in nutritional epidemiology to capture overall dietary patterns. In this study, we combined PCA for dimensionality reduction [33] with K-means++ initialization to enhance cluster stability and reproducibility [34]. PCA facilitated the extraction of dominant dietary variation, while K-means++ reduced sensitivity to random centroid selection and improved the consistency of clustering solutions [22]. This integrated approach allowed the identification of interpretable and well-separated dietary clusters within a large cohort. Rather than emphasizing methodological novelty, our findings suggest that this combination provides a stable and practical framework for characterizing dietary behaviors and examining their associations with clinical outcomes, such as renal function. Based on K-means++ clustering, the non-healthy cluster (cluster 0) was characterized by markedly high intake of sugary drinks [7], along with greater consumption of processed foods [35], instant noodles [36], condiments, organ meats [37], staple foods, and meat [37]. These dietary components have been consistently reported in previous literature to be associated with adverse renal outcomes. In the present study, the K-means++ algorithm demonstrated good performance in classifying dietary patterns, yielding clusters that were both reasonable and interpretable. Moreover, transitions from non-healthy to healthy dietary patterns, as defined by K-means++ clustering, were modestly associated with a lower risk of renal function worsening, despite the relatively small average annual eGFR decline (<1 mL/min/1.73 m^2^) observed across groups. These findings are biologically plausible and support the validity of this approach. Therefore, we suggest that the K-means++ algorithm can be reliably applied to cluster dietary intake, reflecting real-world eating behaviors in a large Taiwanese cohort.

A total of three dietary component clusters were identified using the K-means++ algorithm, each representing distinct nutritional profiles. The cluster characterized by higher intake of processed foods, red meat, and energy-dense items may resemble dietary patterns commonly described as Western-type diets, which have previously been associated with metabolic disorders and kidney function decline. In contrast, the cluster with higher consumption of fruits, vegetables, and plant-based foods shares similarities with prudent or plant-forward dietary patterns, which are generally linked to better cardiometabolic and renal outcomes. These findings are broadly consistent with previous nutritional epidemiology studies conducted in Western and Asian populations, where diets rich in processed foods and animal-based products were associated with increased risk of CKD progression, whereas diets emphasizing plant-based foods were associated with protective renal effects. From our findings, the Non-Health cluster consistently demonstrated the highest intake of sugary beverages, sweets, and processed foods across both time points, whereas the Health cluster was characterized by substantially greater consumption of nutrient-dense food groups. These observations are strongly aligned with previous epidemiological and mechanistic evidence. Intake of sugar-sweetened beverages has been associated with incident CKD and accelerated eGFR decline, as demonstrated in large prospective cohorts including the Atherosclerosis Risk in Communities (ARIC) study and the REGARDS study [38,39]. Diets high in refined sugars further exacerbate insulin resistance and chronic low-grade inflammation, key biological processes contributing to glomerular and tubular injury [7,40,41]. Moreover, processed foods – frequently enriched with sodium and phosphate additives – have been linked to higher blood pressure, increased renal acid load, and glomerular hyperfiltration, thereby accelerating CKD progression [42,43]. In contrast, nutrient-dense dietary patterns – such as the DASH or Mediterranean diet – have been associated with lower levels of inflammatory biomarkers including C-reactive protein and interleukin-6, reduced dietary phosphate burden, improved glycemic control, and slower eGFR decline in multiple cohorts [1,5,44]. Our results therefore align closely with these well-established biological pathways and population-based findings. Importantly, our study extends this evidence base by highlighting that dynamic transitions toward healthier dietary patterns may confer additional renal benefits over time, underscoring the potential clinical relevance of dietary modification in slowing renal function decline. By using a data-driven clustering approach, our study captures real-world dietary behaviors without relying on predefined dietary indices, providing additional insight into how naturally occurring dietary patterns may influence renal function trajectories in a Taiwanese population.

There are several limitations to this study. First, dietary intake was assessed using a semi-quantitative FFQ, which is subject to recall bias and potential misclassification. Second, dietary patterns identified through clustering may act as proxies for broader lifestyle and socioeconomic factors, including education level, health-seeking behavior, and physical activity, which were not comprehensively captured in the present analysis. Therefore, residual confounding related to lifestyle clustering cannot be entirely excluded. Therefore, causal inferences should be interpreted with caution. Third, the generalizability of our findings may be restricted to Taiwanese populations with comparable dietary habits. We noted that cultural dietary habits (e.g., high soy intake, stir-frying methods) might influence the observed associations and that further validation in diverse ethnic groups is warranted to confirm the global generalizability of these findings. Fourth, the overall decline in eGFR during follow-up was modest, which may have attenuated the observed differences between clusters. Finally, we focused exclusively on renal outcomes; other clinically relevant endpoints, including cardiovascular disease and mortality, were not evaluated.

## Conclusions

The K-means++ algorithm reliably identified distinct dietary patterns in a large Taiwanese cohort and revealed that transitions from non-healthy to healthy diets were associated with a reduced risk of renal function worsening. These findings highlight the potential value of dietary modification in preserving renal health and support K-means++ as a robust tool for nutritional epidemiology.

## Supplementary Material

Manuscript clean.docx

graphic abstract version 1.jpg

## Data Availability

Data sharing is not applicable to this article as no data were created or analyzed in this research.
