# Supplementary figures and images for: Impact of dietary component clusters identified by K-means++ on renal function decline in a Taiwanese cohort
Source: Ren Fail. 2026 Jun 1;48(1):2667036. doi: 10.1080/0886022X.2026.2667036 (PMC13231811; doi:10.1080/0886022X.2026.2667036)

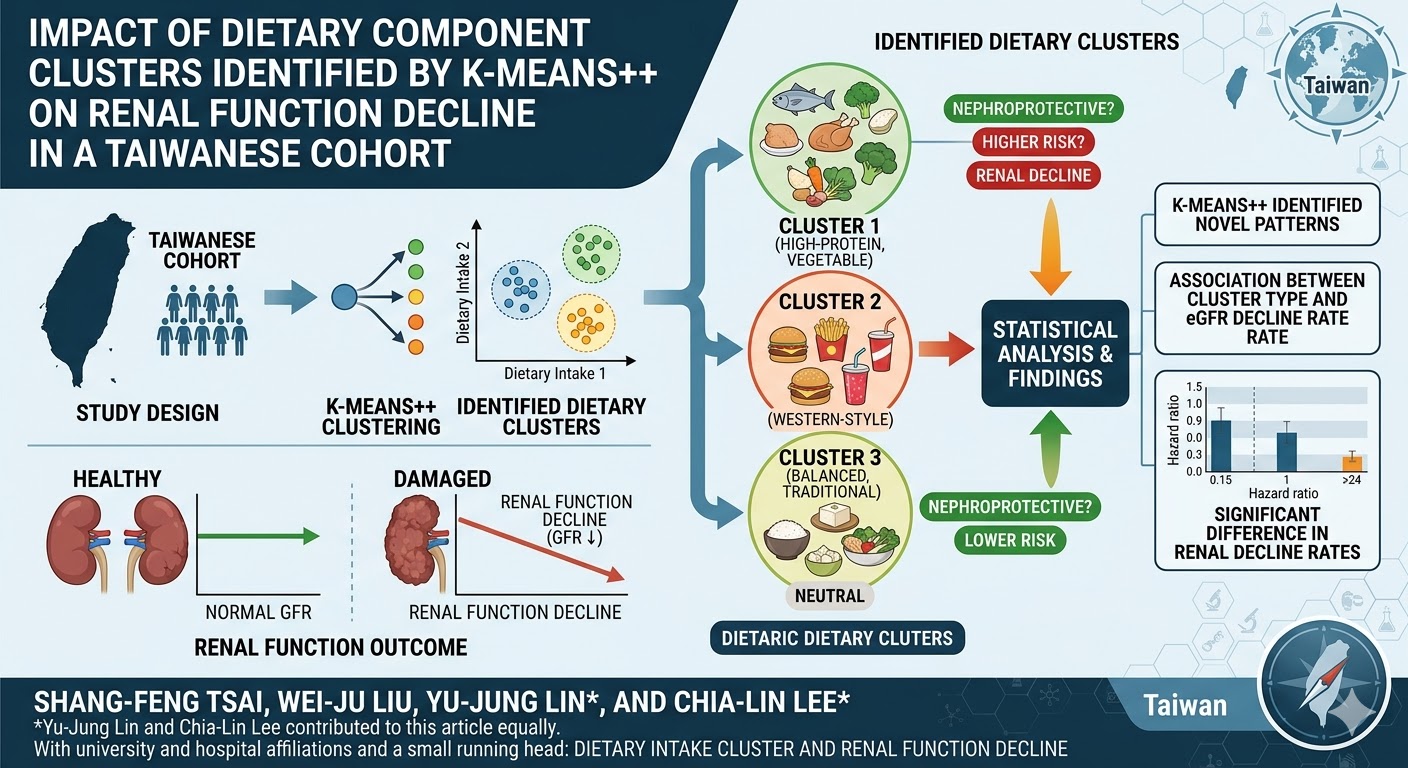

Supplement: graphic abstract version 1.jpg [file IRNF_A_2667036_SM2209.jpg]
